# Gene therapy for primary immune deficiencies: a Canadian perspective

**DOI:** 10.1186/s13223-017-0184-y

**Published:** 2017-02-27

**Authors:** Xiaobai Xu, Chetankumar S. Tailor, Eyal Grunebaum

**Affiliations:** 1 Developmental and Stem Cell Biology, Research Institute, The Hospital for Sick Children, Toronto, ON Canada; 2 Tailored Genes, Toronto, ON Canada; 3 Division of Immunology and Allergy, Department of Paediatrics, The Hospital for Sick Children, Toronto, ON Canada; 4 University of Toronto, Toronto, ON Canada

**Keywords:** Gene therapy, Primary immunodeficiency, Adenosine deaminase deficiency, Canada, Lentivirus, Insertional mutagenesis

## Abstract

The use of gene therapy (GT) for the treatment of primary immune deficiencies (PID) including severe combined immune deficiency (SCID) has progressed significantly in the recent years. In particular, long-term studies have shown that adenosine deaminase (ADA) gene delivery into ADA-deficient hematopoietic stem cells that are then transplanted into the patients corrects the abnormal function of the ADA enzyme, which leads to immune reconstitution. In contrast, the outcome was disappointing for patients with X-linked SCID, Wiskott–Aldrich syndrome and chronic granulomatous disease who received GT followed by autologous gene corrected transplantations, as many developed hematological malignancies. The malignancies were attributed to the predilection of the viruses used for gene delivery to integrated at oncogenic areas. The availability of safer and more efficient self-inactivating lentiviruses for gene delivery has reignited the interest in GT for many PID that are now in various stages of pre-clinical studies and clinical trials. Moreover, advances in early diagnosis of PID and gene editing technology coupled with enhanced abilities to generate and manipulate stem cells ex vivo are expected to further contribute to the benefit of GT for PID. Here we review the past, the present and the future of GT for PID, with particular emphasis on the Canadian perspective.

## Background

Primary immune deficiencies (PID) are a group of inherited immune disorders that can result in predisposition to infections, immune dysregulation, autoimmunity or malignancy. The introduction of newborn screening for severe immune defects as well as better diagnostic modalities and awareness have contributed to increase in identification of PID [1]. Early diagnosis, antibiotic prophylaxis and treatment, immunoglobulin replacement and immunosuppressive medications can help prevent or ameliorate many of the PID manifestations. However, such treatments often require life-long administration and are associated with significant emotional and financial burden to patients, families and society. Moreover, such treatments may lose their effectiveness over time and often do not prevent immune dysregulation disorders or malignancy. Hence, the ultimate cure for most PID requires correction of the defective gene responsible for the immune deficiency.

### Hematopoietic stem cell transplantations for primary immune deficiency

Hematopoietic stem cell transplantations (HSCT) involve the infusion of stem cells typically obtained from bone marrow, peripheral blood or umbilical cord blood to re-establish the hematopoietic and/or immune function. Since the original description of allogeneic bone marrow transplantations for patients suffering from PID in 1968, HSCT have been performed across the world for many severe immune defects [2, 3]. These conditions range from severe combined immunodeficiency (SCID) encompassing all lymphoid lineages such as adenosine deaminase (ADA) deficiency, lymphoid subtypes such as “common” gamma chain (γc) defects to specific T cell defects such as immune dysregulation, polyendocrinopathy, enteropathy, X-linked (IPEX) syndrome. Other PID that can be treated by HSCT include myeloid abnormalities such as Wiskott–Aldrich syndrome (WAS), leukocyte adhesion defect (LAD) or chronic granulomatous diseases (CGD). Throughout the years, HSCT using allogenic human leukocyte antigens (HLA) matched or mis-matched donors have cured thousands of patients with PID. However, allogeneic HSCT are associated with many complications. Chemotherapy is often required prior to HSCT to eliminate the recipient’s residual immune system, which helps prevent rejection of the donor cells. Moreover, many patients experience significant graft versus host (GvH) disease where the donor’s competent immune system recognizes the recipient HLA-expressing cells as foreign and attacks the recipient organs. When HLA-matched or mismatched unrelated donors are used for transplanting patients with PID, which occurs in the majority of HSCT, the risk for GvH disease increases to more than 70% [4]. Although earlier diagnosis, improved infections control, better HLA matching and lesser toxic conditioning regimens are expected to further improve the outcomes of HSCT, such procedures continue to carry significant complications.

### Gene therapy

**Fig. 1:**
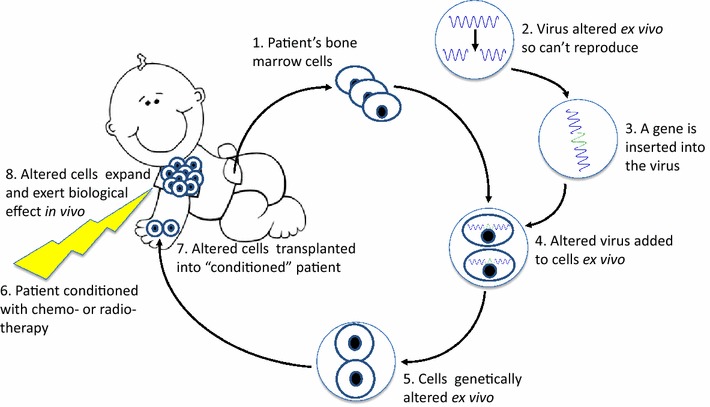
Ex vivo gene therapy. Patient’s cells are collected from bone marrow, peripheral blood or umbilical cord blood (*1*). A virus is altered ex vivo to increase safety and efficacy of gene delivery (*2*). A gene is inserted into the altered virus ex vivo (*3*). The altered virus containing the gene is added to the patient’s cells ex vivo (*4*). The cells are genetically altered ex vivo (*5*). The patient is treated with chemotherapy or radiotherapy (*6*). The genetically altered cells are transplanted into the conditioned patient (*7*). The genetically altered cells expand in the patient and exert their biological effects (*8*)

**Fig. 2:**
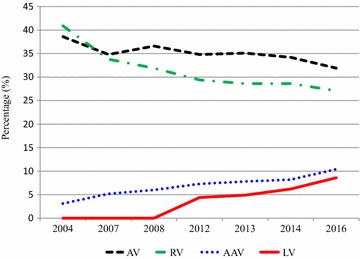
Viral vector use in gene therapy trials worldwide 2004–2016. Viral vectors account for 67% of the total vectors used for gene therapy clinical trials worldwide. The graph depicts the percentage (%) of *AV* adenovirus, *RV* retrovirus, *AAV* adeno-associated virus and *LV* lentivirus vector use of the total viral vectors

Gene therapy (GT), i.e. the use of genetic material to modify cells, has been investigated for numerous conditions since the development of recombinant DNA technology in the late 1970’s. Different forms of GT are being explored, including gene insertion into cells ex vivo that can then be transplanted into the recipient, expand and exert a desired biological effect (Fig. 1) or direct injection of the DNA into the body. GT using ex vivo modified autologous cells could avoid graft rejection and GvHD as the transplanted cells and the recipient have identical HLA. Therefore, it has long been proposed as an alternative treatment to allogeneic HSCT. Different gene delivery systems were developed, which provide either transient or stable gene transfer. Mechanical methods such as liposomes or electroporation can introduce nucleic acids into cells both in vitro and in vivo, albeit at low efficacy while damaging many of the cells. Biological systems, including transposons and viral vectors have been used at increasing frequency to insert genetic material into cells. Viruses now account for 67% of all delivery methods used in GT clinical trials worldwide, with adenovirus and retrovirus vectors representing the majority. Indeed, as data from the “Gene therapy Clinical Trials Worldwide” update from August 2016 (Fig. 2) indicate a steady increase in the use of adeno-associated virus and lentivirus vectors in clinical trials [5]. The viral vectors differ in the size of the genetic material they can harbor as well as their tropism to tissues and cells. Other differences include the virus’ ability to evade the recipient’s immune system, influencing the potential to trigger a neutralizing and possibly harmful immune response. Adenovirus associated virus has been commonly used for the correction of monogenetic disorders in post-mitotic tissues, while retroviral vectors can integrate into the host cell genome. Therefore retroviruses are preferred for the stable gene transfer into proliferating cells, such as hematopoietic stem cells. Indeed, most pre-clinical and clinical GT for PID used members of retroviridae family, including the Murine leukemia virus (MLV) and the human immunodeficiency virus (HIV) of the gamma-retrovirus and lentivirus (LV) genus, respectively. Inserting the gene of interest ex vivo into cells isolated from the patient, which are then given back to the patient, lowers the risk of unwanted effects associated with in vivo delivery, such as ectopic expression of the delivered gene in off-target organs. Furthermore, the therapeutic impact from ex vivo gene delivery is more robust since the gene-based correction is not subject to metabolic or renal clearance and is less likely to trigger immune responses. In some protocols, ex vivo GT may even allow for selection, expansion and quality control of the modified cells before reinfusion, thereby further improving safety and efficacy [6].

The use of LV vectors raised safety and ethical concerns about the possibility of transmitting or promoting HIV infection, including generation of replication competent LV during vector production or mobilisation of the vector by endogenous retroviruses in patients’ genomes. Some of the strategies developed to mitigate the risk for patients and the medical teams include limiting to the number of accessory viral genes, splitting the viral components to different plasmids, reducing the number of viral particles used for transduction and incorporating self-inactivating (SIN) vectors. Nevertheless, close monitoring of patients who receive LV GT is warranted. Importantly, despite the increasing use of LV, there have not been any reports of accidental development of HIV.

### Gene therapy for primary immune deficiencies

GT has been a particularly attractive option for PID. The genes responsible for many PID have been identified, the diseases have been often fatal at a young age and current treatments have commonly been unsatisfactory. Moreover, the success of HSCT and spontaneous reverse mutations that corrected PID supported the hypothesis that autologous GT would be beneficial for such patients. Indeed, PID was the first human condition treated with GT and continues to be in the forefront of such attempts. Ideally, patients should be treated as early as possible before suffering significant infections and organ damage, and when the potential for immune reconstitution is maximal. Specific indications and inclusion criteria vary in accordance to the condition and the protocol. The subsequent discussion will detail the past current and future status of PID GT, with emphasis on Canadian experience and contributions.

## Main text

### Gene therapy for adenosine deaminase

Adenosine deaminase (ADA) is a ubiquitous enzyme that is crucial for the metabolism of adenosine and 2-deoxyadenosine. Impaired function of ADA leads to accumulation of purine metabolites that are particularly toxic to the rapidly proliferating bone marrow cells and thymocytes. Inherited ADA defects account for 15–20% of all causes of SCID, and some Canadian populations such as the Mennonite and Canadian First Nations seem to have increased frequency of ADA deficiency [7]. The implementation of newborn screening (NBS) for severe immune deficiency, and the inclusion of ADA among the conditions tested in some of the NBS algorithms, such as the one implemented in Ontario, are expected to reveal the true incidence of ADA deficiency. T, B and Natural Killer (NK) cells dysfunction is often present in ADA-deficient patients in infancy. In addition, patients may suffer from alveolar proteinosis [8], diverse neuro-developmental abnormalities [9] as well as bone and cartilage malformations [10]. Since the mid-1980, weekly injections of polyethylene glycol-modified bovine ADA (PEG-ADA) have been used to remove the toxic purine metabolites, improve T and B cell functions and correct for some of the non-immunologic abnormalities [11]. However, PEG-ADA does not cure ADA deficiency, as it is effective in only 80% of patients and the immune recovery often diminishes over time [12]. Moreover, the cost of PEG-ADA treatment (>$US 100,000/year) restricts its availability for many patients. Nevertheless, health Ministries of some Canadian provinces, such as Ontario and British Columbia have reluctantly sponsored PEG-ADA treatment. HSCT from an unaffected HLA identical sibling donor without any chemotherapy has been shown to restore immunity in ADA deficient patients, and is currently considered the treatment of choice [13]. However, HLA identical sibling donors are available for only a minority of patients while the use of HLA-mismatched related or unrelated donors is associated with significant morbidity and mortality [14]. These disappointing results of HSCT, together with the early availability of the ADA gene sequence, prompted the investigation of GT for ADA deficiency.

Initial GT trials for ADA deficiency, performed already in early 1990, delivered the ADA gene into T lymphocytes or umbilical cord blood/bone marrow progenitor cells using the murine gamma-retroviral vector. Similar to the protocols used for allogeneic HSCT, autologous GT were done in ADA-deficient patients without the use of cytoreductive conditioning. This was based on the assumption that ADA-proficient cells would have a “survival advantage” over the original ADA-deficient cells. Yet, for ethical reasons, patients enrolled in this trial were given PEG-ADA, which negated the survival advantage of the gene-corrected cells. Hence, despite detection of ADA-corrected cells in the host, inadequate amount of cells persisted to confer significant clinical benefit. It took almost a decade until the group in Italy, led by Drs. Aiuti and Naldini reintroduced non-myeloablative doses of busulfan or melphalan without PEG-ADA, into the GT trials for ADA deficiency [15]. Together with improved gene transduction techniques and the use of MLV-derived replication-deficient vector to deliver the ADA cDNA into cells, the Milan group was able to achieve lasting ADA expression in cells. This resulted in humoral and cellular immune reconstitution, decrease in susceptibility to infections [16] and correction of the bone abnormalities [17]. One Canadian patient who participated in this study is now almost 10 years after receiving GT and is clinically well. Subsequent studies at Great Ormond Street in the UK, at The National Human Genome Institute, the Children’s Hospital Los Angeles, and later the UCLA Mattel Children’s Hospital as well as Japan demonstrated the critical role of non-myeloablative pre-transplantation conditioning in gene therapy for ADA SCID [18–20]. Recently, long-term follow-up (range, 2.3–13.4 years) of the 18 ADA-deficient patients who received ADA GT in Milan revealed that all survived [21]. PEG-ADA was resumed in 3 patients, of which 2 later received HSCT from HLA identical sibling donors that were not available prior to GT. The relatively short follow-up of the ADA-deficient patients who received GT in England and North America precludes direct comparison with the Milan outcome, yet the overall results and safety of all these studies are encouraging. Indeed the success of the Milan ADA GT led to commercialization of the viral vector by GlaxoSmithKline (GSK) as Strimvelis™, which recently received marketing authorization in Europe. The impact of such move on ADA GT practicalities, including cost for patients and availability in North America are still not clear. Impressively, and in contrast to GT trials for other PID described below, all ADA-deficient patients who received GT in the USA and Europe survived, and none experienced abnormal clonal expansions or leuko/lympho-proliferative disorders. Although analyses of retroviral vector integrations in patients’ cells demonstrated insertion near proto-oncogenes sites (including LMO2) similar to those found in other PID trials, there was no skewing of the T cell repertoire or clonal selection/expansion in vivo. Despite the lack of insertional genotoxicity with gamma-retroviruses in ADA GT, concerns regarding leukemogenesis have led to the development of SIN LV vectors. Studies using these vectors for ADA deficiency are currently being completed in England (ClinicalTrials.gov Identifier: NCT01380990) and the USA (ClinicalTrials.gov Identifier: NCT01852071). Deletion of proteins from the vector packaging plasmids and the SIN mechanism have made their use safer. Moreover, as LV vectors can transduce non-dividing cells, such as quiescent hematopoietic stem cells, it is postulated that the efficacy of gene delivery into the very early stem cells will be improved. Interestingly, based on experiments in murine models, the current ADA SIN LV trials continue the administration of PEG-ADA for 30 days after the GT. More than 30 ADA-deficient patients have been treated with the SIN LV vector. Immune reconstitution has been achieved with no vector-related complications, although follow-up period for most patients is still short (<3 years). Several ADA-deficient patients from Quebec and Ontario, who lacked HLA-matched sibling donors, have already received GT under this protocol. Although GT is still very expensive (more than $ US 200,000/patient), the cost is less than the life-long continuation of PEG-ADA and possibly even less than an HLA-mismatched HSCT that is often associated with prolonged admissions and complications. Accordingly, the Ministry of Health in several Canadian provinces have approved the out-of-county expenses. After the control of infections and PEG-ADA administration, and coordination by the Canadian referring team with the centers performing the GT, patients typically spend 7–10 days at the GT center. During this period, the patients’ bone marrow cells are harvested, CD34 expressing cells are selected and transduced with the viral vector, busulfan is administered, and the gene-corrected cells are infused. Patients who are clinically well can return to the referral center prior to the development of chemotherapy-induced neutropenia. Close monitoring and frequent follow-ups are coordinated between the referring teams and the GT centers. In the future, shipping the patients’ bone marrow to designated centers might prevent the need for them to commute, further simplifying GT and reducing its costs. Indeed, researchers in the US and UK have began investigating the effects of cryopreservation of the cells on the success of LV GT for ADA-deficient patients (NCT02999984).

### Gene therapy for common gamma chain

The interleukin-2 receptor gamma subunit (*IL2R*) gene on the X-chromosome encodes for the gamma chain (γc). The chain is a component for intracellular signaling of the IL-2, -4, -7, -9, -15, and -21 receptors, thus it is essential to the development and function of T, B and NK cells. Inherited defects in the γc are the most common cause of SCID in some medical centers [22], although not in others [23]. The male patients tend to present during infancy with recurrent and opportunistic infections such as *Pneumocystis jiroveci* pneumonia, unremitting candida and failure to thrive. The majority of patients lack T cells, yet expansion of B cells may prevent the characteristic lymphopenia. In recent years, with the introduction of NBS for SCID in most US states, as well as some Canadian provinces and European countries, IL2Rγ-deficient patients are being diagnosed earlier, prior to the development of infections.

Similar to other forms of SCID, HSCT can cure the immune defect caused by the impaired γc signaling. The best outcome, with >90% survival and excellent immune reconstitution can be achieved with the use of an HLA-identical sibling donor. Such transplants are typically done without any chemotherapy preparation. HSCT using HLA matched unrelated donors result in >80% survival and long-term immune reconstitution [4]. In recent years, improved outcome has also been reported with the use of HLA mismatched family donors, although immune reconstitution might be delayed and incomplete [24]. Hence, GT has been proposed as an alternative management option for patients without a suitable donor, particularly if patients also have active infections.

Gene therapy trials for X-linked SCID opened in 1999 and 2001 in Neckar, France and Great Ormond Street, UK, respectively. Both sites used autologous CD34+ cells that were transduced ex vivo with a murine gamma-retroviral vector. Gene-modified cells were returned to patients without cytoreductive conditioning. This led to improved cellular and humoral immunity, and patients were able to combat typical childhood infections and resume normal growth and development [25, 26]. However, 5 of the 20 patients treated at these centers developed T cell acute lymphoblastic leukemia 2.5–6 years after GT. The leukemic transformation was attributed to a predilection of the gamma-retroviral vector to integrate near oncogenes. The uncontrolled expression of a cytokine receptor important for the proliferation of T cells might also have contributed to the malignant transformation. While the overall outcome of these initial studies demonstrated that GT for the γc is possible, the significant concerns for safety halted clinical trials of GT for this condition (and others) for several years. Subsequently, modifications were made to the original gamma-retroviral vector to improve its safety, including creation of a SIN construct and replacement of the promoter. Results of GT with the modified construct used in 9 boys in parallel European and US trials, still without preparative conditioning, were recently reported [27]. One patient died from a preexisting adenoviral infection prior to immune reconstitution, while 7 of the 8 surviving patients had functional T cells and were free of infections. Four additional patients have received GT under this SIN gamma-retrovirus protocol (ClinicalTrials.gov Identifier: NCT01129544), which is close to completion. Integration analysis demonstrated no clonal skewing and none of the patients have developed malignancy, yet the follow-up period is still short. Similar to the trend in other PID, GT for X-linked SCID has recently shifted to the use SIN LV. A clinical trial using a codon-optimized SIN LV vector controlled by the ubiquitous elongation factor 1α promoter is being conducted in the US (ClinicalTrials.gov Identifier: NCT01306019). Interestingly, intermediate doses of busulfan were chosen for conditioning patients prior to GT. Initial results of this trial have already been published [28]. The 2 older subjects (aged 24 and 23 years respectively) cleared pre-existing viral infections and were able to stop immunoglobulin infusions. One patient died from pulmonary hemorrhage 27 months after GT while the other patient is clinically well 3 years after GT. Three younger patients (7–15 years old) were also treated, but conclusions regarding the safety and efficacy of this protocol cannot be drawn since their follow-up period is less than 1 year. In 2017, Boston Children’s and several collaborators are expected to open another trial with a SIN LV vector that will also involve administration of low dose busulfan in order to generate IL2Rγ-expressing B cells and to correct the humoral immunity. Future studies comparing the survival, complications and long-term immune reconstitution following HSCT from different donors and GT, with and without conditioning, will enable better assessment of the various treatment options for patients with X-linked SCID.

Using targeted genome editing by artificial nucleases is another interesting approach, although it is still in pre-clinical stages. These include the zinc finger nucleases (ZFNs), the transcription activator-like effector nucleases (TALENs) and the RNA-guided clustered regularly interspaced short palindromic repeats (CRISPR/Cas) nucleases that can efficiently and specifically cause a DNA break at a preselected site. Using ZFN and tailoring of delivery platforms and culture conditions, Naldini’s group were able to target a corrective cDNA into the IL2Rγ gene of stem cells from a patient with X-linked SCID, which led to normalization of hematopoiesis and generation of functional lymphoid cells [29].

### Gene therapy for Wiskott Aldrich Syndrome

The *WAS* gene on the X chromosome encodes for the cytoplasmic WAS protein that affects actin polymerization in hematopoietic cells. WAS protein is important for leukocyte migration and formation of the immunologic synapse. WAS is characterized by increased susceptibility to infections, eczema, as well as the bleeding caused by the thrombocytopenia with platelets of low size and impaired function [30]. Patients also suffer from diverse autoimmune manifestations that may further contribute to the development of thrombocytopenia, vascular abnormalities and malignancies. Antibiotics and immunoglobulin prophylaxis as well as platelet transfusions and immune suppression may provide temporary relief for affected patients, yet most patients eventually develop life-threatening complications. Despite supportive care, the median life expectancy of patients is markedly reduced. Predicting outcome for specific patients based on the mutation, protein expression or clinical grading have been challenging, hence in most cases there should be an attempt to cure the disease. HSCT for WAS have been performed for almost 50 years, with excellent results, particularly if done early in life, with a well matched donor [31]. Indeed, the London group reported 100% survival rate in 34 patients treated between 1996 and 2016 using a variety of graft sources and tailored preparative regimens [32]. Nevertheless, GT is an attractive option for patients already harboring infections such as CMV, suffering from significant co-morbidities or lacking suitable HSCT donors.

The first WAS GT trial was performed between 2006 and 2009 in Munich, Germany and included 10 patients with severe phenotype. Patients received low doses of busulfan followed by transfusions of autologous CD34+ cells transduced with a WASP-expressing gamma-retroviral vector. GT reconstituted T cell function and antibody production. Platelets size normalized and their numbers increased, albeit often remaining below normal range, with resolution of hemorrhagic diatheses [33]. Yet, between 14 months and 5 years after gene therapy, 7 patients developed acute leukemia. Similar to the findings in X-linked SCID GT, the increased tendency of retroviruses to integrate near oncogenes, such as LMO2, was the probable reason. Subsequently, there have been 3 GT trials in Italy, the USA as well as France and England using a SIN LV and the endogenous WAS promoter [34–36]. As of April 2016, 8 patients received GT for WAS following reduced intensity conditioning in Milan, Italy (ClinicalTrials.gov Identifier: NCT01515462). At a median follow-up length of 3.8 years (range: 0.6–5.9), they are all alive and well. After immune reconstitution, marked reduction in severe infection rate was observed and 5 patients were able to stop immunoglobulin supplementation. There was a noticeable decrease in moderate-severe bleeding frequency and all patients became platelet transfusion independent, although platelet numbers have remained below normal. Importantly, no abnormal clonal proliferations were observed [37]. Seven patients suffering from WAS received GT in France (ClinicalTrials.gov Identifier: NCT01347346) and England (ClinicalTrials.gov Identifier: NCT01347242). At the time of the last reported follow-up, 6 were alive with no severe bleeding episodes, and were free of infections and leukemic events. One patient died 7 months after GT due to preexisting, refractory herpes virus infections. GT was also beneficial in a 30 year old patient with severe WAS manifesting with multiple inflammatory complications and lympho-proliferation who required long-term immunosuppressive treatment for disease control [38]. Another LV GT trial for WAS at Boston, USA (ClinicalTrials.gov Identifier: NCT01410825) has enrolled two patients who are reported to have improved immune and hematologic parameters without genotoxicity at early time points. The trials in France, UK and USA are currently recruiting patients. It is expected that the results from these autologous GT studies will enable better comparison with those of allogenic HSCT, including susceptibility to autoimmunity that has been frequently reported following partial correction of WAS [39].

### Gene therapy for JAK3 deficiency

The JAK3 protein kinase delivers signalling into the cells following stimulation of the γc. Apart from the inheritance, which is autosomal recessive in JAK3 deficiency, patient often display a SCID phenotype similar to that seen in γc deficient patients. A single JAK3-deficient patient who failed HSCT received retroviral GT, however the results were only published in abstract form. Disappointingly, there was no evidence of immune reconstitution at 7 months posttreatment [40]. This clinical trial was stopped following the occurrence of leukemia in X-linked SCID GT.

### Gene therapy for chronic granulomatous disease

CGD is caused by impaired function of the NADPH oxidase complex that is important for the production of reactive oxygen species in phagocytes. Consequently, patients are susceptible to infections by catalase-positive microorganisms such as *Staphylococcus aureus*, *Nocardia* spp, *Serratia marcescens, Burkholderia cepacea and Salmonella* spp as well as *Aspergillus* species. Infected areas typically include the lung, lymph nodes, liver, bones and skin. Dysregulated immune responses often result in granuloma formation and other inflammatory disorders involving the bowel. The most common form of CGD is caused by defects in the in the X-linked CYBB gene, which encodes gp91phox. Recognizing the poor long-term prognosis of patients with CGD, particularly those with markedly reduced neutrophil oxidative burst [41] and improvement in transplantation techniques have led to increasing numbers of patients who have benefited from HSCT [42]. Nevertheless, since patients often experience graft rejection or develop GvH disease and inflammatory exacerbations, GT for the X-linked CYBB gene defects has been explored. The first GT trial for CGD used a gamma-retroviral vectors to deliver human gp91phox. However, only few gene-corrected cells persisted, possibly because no pre-GT conditioning was given. Three patients who received GT at National Institute of Health (NIH) following reduced intensity conditioning showed slightly better engraftment and some clinical improvement; However 1 patient died 6 months after GT from a fungal infection [43]. GT performed in Germany involving 2 patients with CGD, using a similar vector, albeit with a different transcriptional control, led to the correction of 15% of the neutrophils shortly after treatment. Unfortunately, both patients developed fatal myelodysplasia secondary to insertional mutagenesis [44]. Similar complications were also noted in 2 additional children with CGD treated in Switzerland, and the patients were rescued with HSCT. Patients with CGD also received unsuccessful GT in London (4 patients) and Seoul (2 patients). To improve efficacy and safety of GT for CGD, SIN gamma-retroviral vector and a LV vector expressing gp91phox were developed with preparative conditioning regimens that are effective and well tolerated. Moreover, the newer constructs carry myeloid-specific promoters and/or allow for post-transcriptional down-regulation of expression in hematopoietic stem cells. Currently 3 US sites (NIH, Boston and Los Angeles) are recruiting patients with CGD who are 23 months or older for a trial with a 3rd generation SIN LV, which directs gp91phox expression from a codon-optimized form of the CYBB gene preferentially to myeloid cells (ClinicalTrials.gov Identifier: NCT02234934). Similar studies using LV are being conducted in Frankfurt, London and Zurich (ClinicalTrials.gov Identifier: NCT01855685), while the site in Paris is also accepting younger children (ClinicalTrials.gov Identifier: NCT02757911). So far, a single child with CGD and invasive liver, brain, abdominal, and pulmonary infections, and inflammatory complications received GT in Europe. He was reported to be stable for 3 months post GT, but then developed fatal respiratory complications [45].

### Gene therapy for leukocyte adhesion defect

LAD is characterized by delayed separation of the cord, neutrophilia, severe gingivitis and periodontitis, and recurrent, cutaneous, non-healing wounds lacking puss formation. Patients commonly suffer from severe recurrent systemic bacterial infections. The classical form of LAD is caused by defects in the CD18 gene, also known as the beta-2 subunit of the leukocyte integrin family or ITGB2. Allogeneic HSCT are the only definitive cure for LAD, but complete donor engraftment has been difficult to achieve [46]. Two patients with severe LAD received RV mediated CD18 gene-corrected stem cells. After the infusion, only 0.1% of the patients’ neutrophils expressed CD18, and these cells disappeared within 2 months of GT. Subsequent GT for LAD has been restricted to animal models [47].

### Gene therapy for other PID

GT for other PID are currently at various in vitro and in vivo pre-clinical stages (Table 1), often needing to address unique challenges associated with specific diseases.

**Table 1:** Preclinical studies of GT for other PID

Condition	Gene defect	Delivery	Achievements and challenges	References
Recombination activating gene 1 and 2 deficiency	RAG-1 and RAG-2	SIN LV	Insufficient expression in vivo causing Omenn’s syndrome	[48, 49]
Artemis deficiency	DCLRE1C	SIN LV	Artemis deficient- mice and human stem cells differentiated in vivo into functional T and B cells	[50]
CD3gamma deficiency	CD3γ	Retro	Constitutive IL-2 synthesis	[51]
Reticular dysgenesis	AJ2	SIN LV	Limited in vitro data	[52]
Purine nucleoside phosphorylase deficiency	PNP	SIN LV	Transient effect in vivo	[53]
ZAP70 deficiency	ZAP70	LV, electroporation	Direct delivery into the thymus	[54, 55]
MHC class II deficiency	CIITA	RV	No recent studies. Concerns about autoimmunity	[56]
HyperIgM syndrome	CD40 ligand	SIN LV TALEN	Targeted CD40 ligand insertion to prevent uncontrolled activation	[57, 58]
IPEX syndrome	FOXP3	LV	Delivery into peripheral T cells	[59]
HLH	PRF	SIN LV	High % of gene corrected cells or high level of perforin expression required in vivo	[60, 61]
XLP	SH2D1A	LV	Incomplete immune reconstitution in vivo, non-physiological expression	[62]
XLA	BTK	TALEN	Corrected mutation and phenotype in vivo	[63]

*SIN* self inactivating, *LV* lentivirus, *RV* retrovirus, *IPEX* immune dysregulation, polyendocrinopathy, enteropathy X Linked, *XLP* X linked lymphoproliferative disease, *XLA* X linked agammaglobulinemia

### The future of gene therapy

In recent years, advances in gene manipulation and vector design are expected to bring GT closer to clinical reality. A major breakthrough is the development of site-specific gene editing tools. By creating site-specific breaks in the DNA near the location of a known mutation, a cell’s natural repair mechanisms can be utilized to incorporate normal segments of DNA. This strategy positions genes in their endogenous locations under the control of normal regulatory elements, thereby decreasing the risk of insertional mutagenesis or ectopic protein expression. GT for PID uses HSC derived from patients’ bone marrow, mobilized peripheral mononuclear cells, or rarely from the recipient’s own cord blood. In the upcoming years, advances in reprograming blood, skin and other tissues into pluri-potent stem cells (iPSC) followed by ex vivo differentiation of these cells into hematopoietic and immune lineages are expected to limit the need for invasive procedures. Another important development is the ability to efficiently freeze, thaw and expand stem cells. This may circumvent the need to send Canadian patients, and families, out of the country to the few locations where gene delivery is currently being performed. Centralizing stem cells manipulation and gene delivery will enable resources and expertise to concentrate at specific GMP facilities, while allowing patients to continue receiving care at local Canadian centers experienced in transplantations. Centralization will also facilitate monitoring for long-term complications secondary to GT and conduction of novel GT trials.

## Conclusions

The use of GT to cure PID is developing rapidly. While there are still significant challenges, the recently improved safety and efficacy measures in GT suggest that such treatment may soon become a standard of care for diverse PID, including many affected Canadian patients.

## Abbreviations

ADA: adenosine deaminase
CGD: chronic granulomatous disease
CRISPR: clustered regularly interspaced short palindromic repeats
γc: gamma chain
GT: gene therapy
GvH: graft versus host
HIV: human immunodeficiency virus type 1
HLA: human leukocyte antigens
HLH: hemophagocytic lymphohistiocytosis
HSC: hematopoietic stem cell
HSCT: hematopoietic stem cell transplantation
IL2Rγ: interleukin-2 receptor gamma
IPEX: immune dysregulation, polyendocrinopathy, enteropathy, X-linked
IPSCs: induced pluripotent stem cells
JAK: Janus kinase
LAD: leukocyte adhesion deficiency
LV: lentivirus
MLV: Murine leukemia virus
NIH: National Institute of Health
PEG-ADA: polyethylene glycol-modified adenosine deaminase
PID: primary immunodeficiency diseases
RAG: recombination activating gene
SCID: severe combined immunodeficiency
SCIDX-1: X-linked severe combined immunodeficiency
SIN: self-inactivation
TALEN: transcription activator-like effector nucleases
WAS: Wiskott–Aldrich syndrome
ZFNs: zinc-finger nucleases
NBS: newborn screening
